# Hospitalization following outpatient medical care for influenza: US influenza vaccine effectiveness network, 2011‐12—2015‐16

**DOI:** 10.1111/irv.12616

**Published:** 2018-12-19

**Authors:** Grace D. Appiah, Jessie R. Chung, Brendan Flannery, Fiona P. Havers, Richard K. Zimmerman, Mary Patricia Nowalk, Arnold S. Monto, Emily T. Martin, Manjusha Gaglani, Kempapura Murthy, Lisa A. Jackson, Michael L. Jackson, Huong Q. McLean, Edward A. Belongia, Alicia M. Fry

**Affiliations:** ^1^ Epidemic Intelligence Service Centers for Disease Control and Prevention Atlanta Georgia; ^2^ Influenza Division Centers for Disease Control and Prevention Atlanta Georgia; ^3^ University of Pittsburgh Schools of Health Sciences and UPMC Pittsburgh Pennsylvania; ^4^ Department of Epidemiology University of Michigan School of Public Health Ann Arbor Michigan; ^5^ Baylor Scott and White Health Texas A&M University Health Science Center College of Medicine Temple Texas; ^6^ Kaiser Permanente Washington Health Research Institute Seattle Washington; ^7^ Marshfield Clinic Research Institute Marshfield Wisconsin; ^8^ Present address: Division of Foodborne, Waterborne and Environmental Diseases Centers for Disease Control and Prevention Atlanta Georgia

**Keywords:** hospitalization, influenza, medically attended acute respiratory illness, outpatients

## Abstract

Over five seasons, we determined the proportion of outpatients with laboratory‐confirmed, influenza‐associated illness who were hospitalized within 30 days following the outpatient visit. Overall, 136 (1.7%) of 7813 influenza‐positive patients were hospitalized a median of 4 days after an outpatient visit. Patients aged ≥ 65 years and those with high‐risk conditions were at increased risk of hospitalization. After controlling for age and high‐risk conditions, vaccination status and infecting influenza virus type were not associated with hospitalization risk among adults.

## 1 INTRODUCTION

Influenza virus infection is associated with a high disease burden. During the 2010‐2011 through the 2015‐2016 influenza seasons, influenza virus infection resulted in a cumulative estimated healthcare burden of 4.3‐16.7 million outpatient medical visits and 140 000‐700 000 hospital admissions in the United States.^1^ A proportion of patients who present for care in the outpatient setting subsequently require hospitalization for more severe illness or influenza‐related complications. There are few estimates of the proportion of patients hospitalized following outpatient care for influenza.^2^ Better characterization of these patients can improve estimates of influenza burden based on hospital surveillance. To better understand the population of patients admitted to the hospital following an influenza‐associated outpatient visit, and explore risk factors for hospitalization among outpatients with influenza, we evaluated data from persons who sought outpatient care for laboratory‐confirmed influenza over five influenza seasons.

## 2 METHODS

We analyzed data collected from the US Influenza Vaccine Effectiveness (Flu VE) Network over five consecutive influenza seasons, from 2011‐2012 to 2015‐2016. As previously described, the Flu VE Network enrolls patients aged ≥ 6 months seeking medical care within 7 days of illness onset for an acute respiratory illness (ARI) with cough (with cough and/or fever in 2011‐2012) at outpatient healthcare facilities (primary care and acute care setting) associated with study sites in Michigan, Pennsylvania, Texas, Washington and Wisconsin.^3^ In addition, two study sites (Wisconsin and Pennsylvania) enrolled patients meeting inclusion criteria from emergency departments. Patients who reported receipt of one or more doses of influenza antiviral medication within the past 7 days were excluded from enrollment. Nasal and throat swabs were collected at enrollment for influenza virus testing by reverse transcription polymerase chain reaction (RT‐PCR). All patients completed an enrollment interview and provided informed consent for data extraction from medical records. Patients were classified as having a high‐risk condition if they had at least one medical encounter during the year before enrollment associated with an International Classification of Diseases (9^th^ [ICD‐9] or 10th [ICD‐10] revision) diagnosis code corresponding to a high‐risk medical condition as defined by the US Advisory Committee for Immunization Practices (ACIP).^4^

Following enrollment, discharge codes for all hospitalizations and Current Procedural Terminology (CPT) codes for imaging procedures ordered (including chest/sinus x‐ray and computed tomography scan) within 30 days were identified from electronic medical records (Table S1). A total of 98% of CPT codes were for chest imaging and 2% were for sinus imaging procedures. Hospitalizations associated with diagnostic codes for medically attended acute respiratory infection (MAARI) were also identified (Table S2). Multivariable logistic regression was used to calculate adjusted odds ratios and 95% confidence intervals for predictors of any hospitalization among patients aged ≥ 18 years with laboratory‐confirmed influenza within 14 days of enrollment; analysis of risk factors for hospitalizations excluded children aged < 18 years due to low numbers of hospitalizations. We performed a sub analysis of hospitalization among outpatient adults prescribed antiviral medication ≤ 7 days after enrollment and excluded patients hospitalized on the day of or day after enrollment, before one would expect to see the full effect of antiviral treatment. Analyses were conducted in SAS version 9.3 (SAS Institute Inc., Cary, NC).

## 3 RESULTS

Over five influenza seasons, 34 385 patients with ARI were enrolled from primary or acute care settings in the US Flu VE network study and 7813 (22.7%) patients tested RT‐PCR positive for influenza. The mean age of enrolled patients with laboratory‐confirmed influenza was 32.7 years (range, 9 months to 106 years). The majority were non‐Hispanic White (5848; 74.8%), and 4303 (55.1%) were female. Among 2913 children aged < 18 years with laboratory‐confirmed influenza, 567 (19.5%) had high‐risk conditions compared to 1907 (38.9%) of 4900 adults with influenza.

Overall, 136 (1.7%) patients, 25 children and 111 adults, with influenza were hospitalized within 30 days following outpatient enrollment. Among outpatients aged 6 months to 4 years (n = 693) and 5‐17 years (n = 1856) enrolled in primary care or acute care settings, 1.4% and 0.7% were hospitalized; among 168 children enrolled from emergency departments, 13 (8%) were hospitalized. Among children with influenza and high‐risk conditions, 7 of 567 (1.2%) were hospitalized. The majority of hospitalizations within 30 days (81.6% [n = 111]) occurred among adults aged ≥ 18 years enrolled from primary care or acute care settings; among 20 adults enrolled from emergency departments, none were hospitalized within 30 days.

Most hospitalizations (72.8% [n = 99]) occurred within 14 days after outpatient enrollment; hospitalizations within 14 days included the majority (57 [93.4%] of 61) of hospitalizations with MAARI diagnostic codes. The highest proportions of hospitalizations within 14 days occurred among adults aged ≥ 65 years (n = 36 [4.4%] of 819) and adults with ≥1 high‐risk conditions (n = 64 [3.4%] of 1889) (Table 1). The same groups were more likely to have outpatient chest/sinus imaging; 315 (38.0%) of 829 adults aged ≥ 65 years and 599 (24.2%) of 2474 adults with ≥1 high‐risk conditions had an order for chest/sinus imaging (Table 2). Overall, 1239 (15.9%) of 7813 outpatients with laboratory‐confirmed influenza had an order for chest imaging, an indicator of clinical suspicion for pneumonia or more severe respiratory diseases. Among 136 patients with laboratory‐confirmed influenza who were hospitalized within 30 days after seeking outpatient care, 56 (41%) sought outpatient care ≤2 days since illness onset and 59 (43%) sought care 3‐4 days after illness onset. One‐third of hospitalizations occurred on the day of or day after the outpatient visit (Figure S1), and 16 (12%) of those hospitalized were admitted within 2 days of illness onset. Overall, 23 (17%) of 136 hospitalized patients with influenza (3 children and 20 adults) received an antiviral prescription prior to the date of hospital admission; most of these (18 [78%] of 23) received an antiviral prescription >1 day prior to admission.

**Table 1:** Predictors of hospitalization within 14 d following acute respiratory infection (ARI)‐associated outpatient visit among adults with laboratory‐confirmed influenza, US Influenza Vaccine Effectiveness Network, 2011‐2016

Characteristic	Total	Hospitalized	(%)	Adjusted odds ratio (95% CI)
Total	4869	80	(1.6)	
Age group (years)				
18‐49	2661	25	(0.9)	REF
50‐64	1389	19	(1.4)	1.03 (0.55‐1.92)
≥65	819	36	(4.4)	2.43 (1.34‐4.40)
Gender^b^				
Female	2876	50	(1.7)	REF
Male	1991	30	(1.5)	0.81 (0.51‐1.30)
Race/Ethnicity^b^				
White, non‐Hispanic	3936	72	(1.8)	REF
Black, non‐Hispanic	320	2	(0.6)	0.39 (0.09‐1.62)
Other, non‐Hispanic	332	2	(0.6)	0.40 (0.10‐1.66)
Hispanic	270	4	(1.5)	1.07 (0.38‐3.01)
High‐risk condition				
None	2980	16	(0.5)	REF
≥1 condition	1889	64	(3.4)	5.20 (2.86‐9.46)
Vaccination status^a^				
Unvaccinated	2963	31	(1.2)	REF
Vaccinated	2107	48	(2.3)	1.05 (0.63‐5.96)
Season^b^				
2011‐2012	391	4	(1.0)	1.05 (0.35‐3.18)
2012‐2013	1301	23	(1.8)	1.33 (0.72‐2.45)
2013‐2014	928	23	(2.5)	1.91 (0.61‐5.96)
2014‐2015	1361	22	(1.6)	REF
2015‐2016	888	8	(0.9)	0.65 (0.21‐2.05)
Virus type/Subtype^b^				
A/H1N1pdm09	1469	29	(2.0)	2.10 (0.74‐6.02)
A/H3N2	2307	42	(1.8)	1.57 (0.73‐3.41)
B	1002	9	(0.9)	REF

Data are no. (%) or adjusted Odds ratio (95% CI). REF, referent group. All variables were included in the multivariable model. Total excludes 31 adults with laboratory‐confirmed influenza hospitalized 15 to 30 days after enrollment.

Missing for 2 patients.

Missing for 11 patients.

Vaccination status was determined using electronic medical records and plausible self‐report. An additional 69 patients were vaccinated 0‐13 days prior to illness onset.

a Chi‐square test for difference in the proportion hospitalized by season was not statistically significant (p = 0.09).

b 91 patients tested positive for an influenza A virus that was not able to be subtyped.

**Table 2:** Frequency of outpatient chest imaging among adult and pediatric patients with laboratory‐confirmed influenza (N = 7813), Vaccine effectiveness network, 2011‐2016

Factor	Category	N	Chest imaging (%)
Age group (years)	6 mo‐4	791	51 (6.5)
	5‐17	2122	138 (6.5)
	18‐49	2672	405 (15.2)
	50‐64	1399	330 (23.6)
	≥ 65	829	315 (38.0)
Chronic conditions	None	5339	640 (12.0)
	≥ 1	2474	599 (24.2)
Vaccination status	No	4611	628 (13.6)
	Yes	3085	588 (19.1)
Virus type/Subtype	A(H1N1)pdm09	1980	353 (17.8)
	A(H3N2)	3739	605 (16.2)
	Influenza B	1963	266 (13.6)

In the multivariable model, factors independently associated with hospitalization within 14 days of the outpatient visit included age ≥65 years and presence of ≥1 high‐risk condition (Table 1). Vaccination status, season and virus type/subtype were not associated with hospitalization after controlling for age and high‐risk conditions. After excluding adults hospitalized within 1 day of enrollment, receiving an outpatient antiviral prescription was not significantly associated with hospitalization within 30 days of outpatient visit in a regression model including all covariates (data not shown).

## 4 DISCUSSION

Over five influenza seasons, a small percentage (1.7%) of patients with laboratory‐confirmed influenza‐associated ARI were hospitalized within 14 days of the outpatient visit in our study population. This finding was consistent with the low percentage (1%) of hospitalizations among patients enrolled in the 2016‐17 and 2017‐18 seasons (unpublished data). The percentage of outpatients subsequently hospitalized was highest (~4%) for adults aged ≥ 65 years and for patients with ≥1 high‐risk condition (~3%). These findings are supportive of current guidance targeting older adults and persons with underlying high‐risk medical conditions for antiviral treatment in the outpatient setting.^4^ In a large individual patient data meta‐analysis of randomized placebo‐controlled studies, antiviral treatment was shown to reduce hospital admissions among treated outpatients.^5^

Our results are consistent with the low proportions of hospitalizations observed among adults with influenza enrolled in placebo arms of controlled trials assessing the efficacy of oseltamivir.^5^ Similar to these trials, we did not enroll from subspecialty clinics where persons with underlying medical conditions or increased frailty may seek care. One study among a cohort of frail adults with a median age of 75 years suggested that 20% of patients with influenza were hospitalized^6^; thus the risk of hospitalization could be higher in some patient populations. Also, we enrolled only a small number of patients from emergency departments. Thus, our findings should not be extrapolated to all outpatient populations. Among those hospitalized, almost half (46%) were admitted to the hospital on the day of or day after the outpatient visit.

The risk factors for hospitalization that were identified, older age and presence of ≥1 high‐risk conditions, have been previously reported for seasonal influenza.^7^, ^8^ In contrast, during the 2009 H1N1 pandemic, one population‐based cohort study identified younger adults as high‐risk groups for hospitalization and severe illness when infected with H1N1pdm09 virus.^9^ Thus, risk factors for hospitalization may differ during seasons in which a novel virus emerges and needs to be evaluated rapidly to inform interventions.

Our findings are limited by the relatively small number of hospitalizations that occurred among patients with influenza in our study population. Further, hospitalizations occurring after outpatient enrollment may not have been directly related to influenza complications, although most hospitalizations within 14 days of the outpatient visit included a MAARI discharge code. This study lacked statistical power to assess the effectiveness of antiviral prescriptions on subsequent hospitalization and differences in hospitalization rates by vaccination status. We relied on administrative codes to identify patients with high‐risk conditions and orders for sinus and chest imaging. Diagnostic codes for medical encounters resulting in a chest imaging procedure were not available to determine whether the patient had lower respiratory tract infection (LRTI) symptoms.

In summary, hospitalization among influenza‐positive persons seeking care for ARI in general outpatient settings occurred infrequently. However, older adults and those with high‐risk conditions were at higher risk for hospital admission following outpatient care. The true burden of hospitalizations after an outpatient visit is likely different for different patient populations. The finding of increased risk of hospitalization among persons aged ≥ 65 years and those with high‐risk conditions supports current recommendations for prompt, empiric use of influenza antiviral treatment in high‐risk outpatients with suspected or confirmed influenza.

## CONFLICT OF INTEREST

EAB, MG, and HQM have received research funds from MedImmune, LLC. ETM, MPN, and RZ have received research funds from Merck & Co., Inc. LAJ has received research funds from Novavax. ETM has received research funds from Roche Pharmaceuticals. ASM and RZ have received research funds from Sanofi Pasteur, Inc. ASM has received personal fees from Novartis and Protein Sciences Corporation. RZ has received research funds from Pfizer Inc. The remaining authors report no conflict of interests.

## DISCLAIMER

The findings and conclusions in this report are those of the authors and do not necessarily represent the official position of the Centers for Disease Control and Prevention.
