# Supplementary material for: Hospitalization following outpatient medical care for influenza: US influenza vaccine effectiveness network, 2011‐12—2015‐16
Source: Influenza Other Respir Viruses. 2018 Dec 19;13(2):133–7. doi: 10.1111/irv.12616 (PMC6379632; doi:10.1111/irv.12616)
Supplement: Supplementary file 1 [file IRV-13-133-s001.docx]

**Supplementary Appendix**

Title: Hospitalization following Outpatient Medical Care for Influenza: US Influenza Vaccine Effectiveness Network, 2011-12—2015-16

**Supplementary Material:**

**List of Contents:**

Supplementary Figure 1. Time interval between Outpatient Enrollment and Hospital Admission among Adults with Laboratory-confirmed Influenza, Vaccine Effectiveness Network, 2011-2016.

Supplementary Table 1. Current Procedures Terminology (CPT) Codes for Chest/Sinus Imaging Performed during 30 Days Following Outpatient ARI Visit Vaccine Effectiveness Network, 2011–2016.

Supplementary Table 2. Diagnostic Codes for Medically Attended Acute Respiratory Infection (MAARI) during 30 Days Following Outpatient ARI Visit Vaccine Effectiveness Network, 2011–2016.

**Supplementary Figure 1.** Time interval between Outpatient ARI Visit and Hospital Admission among Adults with Laboratory-Confirmed Influenza, Vaccine Effectiveness Network, 2011-2016

**Supplementary Table 1.** Current Procedures Terminology (CPT) Codes for Chest/Sinus Imaging Performed during 30 Days Following Outpatient ARI Visit Vaccine EffectivenessNetwork, 2011–2016

| **CPT Code** | **Procedure Description** |
| --- | --- |
| 70210 | X-RAY EXAM OF SINUSES |
| 70220 | X-RAY EXAM OF SINUSES |
| 70486 | COMPUTED TOMOGRAPHY (CT) SINUS |
| 71010 | RADIOLOGIC EXAMINATION, CHEST; SING |
| 71015 | RADIOLOGIC EXAMINATION, CHEST; STER |
| 71020 | RADIOLOGIC EXAMINATION, CHEST, TWO VIEWS, FRONTAL and LATERAL |
| 71021 | RADIOLOGIC EXAMINATION, CHEST, TWO VIEWS, FRONTAL and LATERAL WITH APICAL LORDOTIC PROCEDURE |
| 71022 | RADIOLOGIC EXAMINATION, CHEST, TWO VIEWS, FRONTAL and LATERAL WITH OBLIQUE PROJECTIONS |
| 71023 | RADIOLOGIC EXAMINATION, CHEST, TWO VIEWS, FRONTAL and LATERAL WITH FLUOROSCOPY |
| 71030 | RADIOLOGIC EXAMINATION, CHEST, COMPLETE, MINIMUM OF 4 VIEWS |
| 71034 | RADIOLOGIC EXAMINATION, CHEST, COMPLETE, MINIMUM OF 4 VIEWS WITH FLUOROSCOPY |
| 71035 | RADIOLOGIC EXAMINATION, CHEST, SPECIAL VIEWS |
| 71100 | RADIOLOGIC EXAMINATION, RIBS, UNILA |
| 71101 | RADIOLOGIC EXAMINATION, RIBS, UNILA |
| 71110 | RADIOLOGIC EXAMINATION, RIBS, BILAT |
| 71111 | X-RAY EXAM OF RIBS/CHEST |
| 71260 | CT CHEST, GENERAL |
| 71270 | CT CHEST, HIGH RESOLUTION |

**Supplementary Table 2.** Diagnostic Codes for Medically Attended Acute Respiratory Infection (MAARI) during 30 Days Following Outpatient ARI Visit Vaccine Effectiveness Network, 2011–2016.

| **Diagnostic Code** | **Description** |
| --- | --- |
| 487, 488 | Influenza code |
| 480, 483, 484, 485, 486 | Pneumonia code |
| 382, 382.4, 382.9 | Otitis media code |
| 79.99 | Unspecified viral illness code |
| 460 | Nasopharyngitis code |
| 462, 463, 464 | Throat-related ARI code |
| 461 | Sinusitis code |
| 465 | Unspecified ARI code |
| 786.07 | Wheezing code |
| 466, 490, 491 | Bronchitis or bronchiolitis code |
| 493 | Asthma code |
| 780.60 | Fever, unspecified code |
| 786.2 | Cough code |
